# The Multifaceted Roles of Zinc Finger Proteins in Pluripotency and Reprogramming

**DOI:** 10.3390/ijms26115106

**Published:** 2025-05-26

**Authors:** Yiwei Qian, Qiang Wu

**Affiliations:** The State Key Laboratory of Quality Research in Chinese Medicine, Faculty of Chinese Medicine, Macau University of Science and Technology, Macau, China; tiffanyqyw@163.com

**Keywords:** zinc finger proteins, pluripotent stem cells, differentiation, reprogramming

## Abstract

Zinc finger proteins (ZFPs) play a crucial role in regulating gene expression. In recent years, there has been increasing evidence highlighting the importance of zinc finger proteins in pluripotent stem cells, which hold great promise in regenerative medicine. The general mechanism by which zinc finger proteins function in gene regulation of pluripotent stem cells involves their interaction with core transcriptional regulatory networks. ZFPs can either enhance key pluripotency genes to maintain pluripotency or promote differentiation of stem cells towards specific lineages by suppressing these key pluripotency genes. Hence, understanding the role of ZFPs in pluripotency and reprogramming is crucial for unraveling the complex regulatory network that governs cell fate decisions. Here we provide a comprehensive review of the current knowledge regarding the multifaceted role of ZFPs in pluripotency maintenance and reprogramming. We propose that more efforts should be focused on fully understanding the fascinating functions of ZFPs in stem cell fate decision.

## 1. Introduction

The zinc finger domain was first identified in 1985 as a repeated zinc-binding motif in *Xenopus* transcription factor IIIA (TFIIIA), which contains conserved cysteine (Cys) and histidine (His) ligands [1]. In 1988, Frankel et al. elucidated that zinc finger proteins (ZFPs) consist of a compact amino acid sequence, where conserved Cys and His residues that self-fold by binding Zn^2+^ form short, stable, finger-like structures called zinc fingers [2]. Based on sequence, structural, and functional attributes, ZFPs are classified into nine types, namely C2H2, C4, C6, C8, C2HC5, C3HC4, C2HC, C3H, and C4HC3 [3]. Additionally, Krishna et al. categorized these proteins into eight groups according to their distinct spatial configurations, including the C2H2 like, Gag knuckle, Treble clef, Zinc ribbon, Zn2/Cys6, TAZ2 domain like, Zinc binding loops, and Metallothionein [4]. ZFPs contain a tandem of zinc finger motifs, which usually serve as interactors binding to RNA, DNA, small molecules, or proteins [5]. Among the various DNA-binding motifs, classical ZFPs form the largest family of sequence-specific DNA-binding proteins. This family is defined by the sequence CysX_2–5_CysX_12–18_HisX_3–5_His, where X represents an arbitrary amino acid [6]. Thus, ZFPs greatly expand their diverse role in gene regulation under different cellular environments or stimuli through different combinations of multiple zinc finger motifs.

As pivotal eukaryotic transcription factors, ZFPs function as either activators or repressors of gene expression [7,8,9], leveraging their versatile binding modes and affinities to orchestrate critical biological processes such as embryonic development [10,11]. Notably, ZFPs emerge as master regulators of pluripotency and lineage specification in pluripotent stem cells (PSCs), including embryonic stem cells (ESCs) [12,13]. By establishing intricate gene regulatory networks, these proteins maintain PSC self-renewal [14] while guiding the cells towards specific lineages during differentiation [15]. Given their central role in PSC fate decisions, deciphering the universal principles governing ZFP-mediated gene regulation and their multifaceted functions in stem cell biology is imperative for advancing regenerative medicine and unlocking the therapeutic potential of PSCs. We review our current understanding of ZFP regulatory mechanisms and their diverse roles in PSCs, providing a framework for future investigations in this rapidly evolving field.

## 2. ZFPs and the Transcriptional Regulatory Networks of PSCs

PSCs possess two important properties: First, they can self-renew indefinitely in the long term; second: they are pluripotent and have the potential to differentiate into almost all cell types [16]. PSCs encompass various cell types, including embryonal carcinoma (EC) cells, mouse embryonic stem cells (mESCs), human embryonic stem cells (hESCs), pluripotent cell lines derived from germ cells, and induced pluripotent stem cells (iPSCs) [17]. At different stages of embryo development, three types of stem cells can be established through different culture systems: ESCs, extraembryonic endoderm cells (XENs), and trophoblast stem cells (TSCs) [18,19]. PSCs constantly balance self-renewal with differentiation, a process influenced by transcriptional regulatory networks, which together dictate cell fate decisions [20].

**Figure 1:**
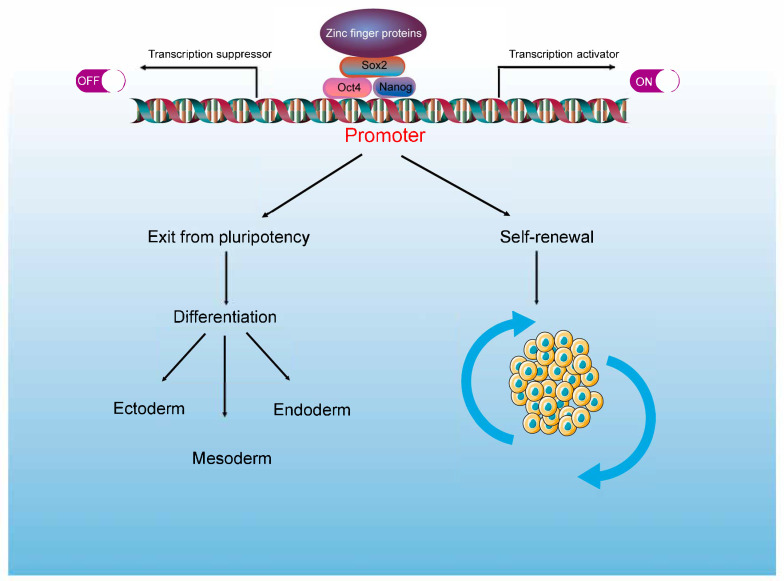
An overview of the roles of ZFPs in transcriptional regulatory networks. ZFPs collaborate with key transcription factors (TFs) of the core transcriptional network, acting as either transcriptional activators or suppressors, thus regulating the self-renewal capacity of PSCs or triggering the exit of pluripotency and PSC differentiation.

Research demonstrates that Oct4 (Pou5f1), Sox2, and Nanog form a core transcriptional factor (TF) network whose precise expression sustains pluripotency, while dysregulation disrupts self-renewal and triggers differentiation [21]. ZFPs act as versatile regulatory factors, exerting critical roles in maintaining PSC identity through their unique dual functions of transcriptional activation and repression [7,8,9] (Figure 1). For example, joint analysis of microarray datasets (GSE30293) and ChIP–chip data has unveiled that Zfp281 exhibits dual functional roles as both a transcriptional activator and repressor [7,8]. ChIP experiments further identified two distinct Zfp281 binding sites within the *Nanog* promoter region. Zfp281 not only directly activates *Nanog* expression by binding to a regulatory element adjacent to the Oct4–Sox2 binding site [7] but also restricts *Nanog* transcription under specific conditions [8]. Additionally, Zfp281 recruits AFF3 to the *Meg3* enhancer region of the *Dlk1–Dio3* locus, facilitating transcriptional elongation [22]. Prdm14 maintains stem cell identity through dual regulatory mechanisms: It suppresses the transcription of ExEn lineage-specific differentiation genes while promoting the activation of specific core ESC maintenance genes [9]. Moreover, ZFPs affect transcriptional regulation of downstream target genes via various functional domains. For instance, CIBZ (ZBTB38) exerts its transcriptional repression functions via the BTB domain [23] and plays a role in G1/S transition partly depending on Nanog expression [24]. Similarly, Sall4 plays an essential role in controlling the pluripotent property of ESCs by binding to AT-rich regions of genomic DNA [25,26]. Furthermore, the role of Sall4 extends to gene suppression by forming complexes with silencer entities such as nucleosome remodeling and deacetylase (NuRD) [27] and gene activation by interacting with pluripotent factors Oct4, Nanog, and Sox2 [28,29,30]. In summary, ZFPs play a crucial role in affecting the core transcriptional regulatory network, and their regulatory roles in pluripotency maintenance of PSCs will be elaborated on in subsequent sections.

## 3. ZFPs and Epigenetic State of PSCs

### 3.1. ZFPs and DNA Methylation

ZFPs play pivotal roles in the DNA methylation regulatory network through diverse mechanisms, forming an intricate interplay between their target-binding specificities and precise epigenetic modulation. Firstly, ZFP57 employs its first two C_2_H_2_ zinc finger domains to recognize methylated CpG sites (TGC^met^CGC) [31] and cooperatively recruits DNA methyltransferases via KAP1 to maintain the DNA methylation imprint [32] while concomitantly regulating H3K9me3 deposition on both imprinted and non-imprinted regions of the maternally inherited chromosome in mESCs during preimplantation development [33]. Furthermore, Zbtb34 competitively binds telomeric DNA through its zinc finger domain, and its upregulation in mESCs significantly promotes telomere elongation while enhancing cellular proliferative capacity [34]. Moreover, ZBTB2 acts as a reader of unmethylated DNA in mESCs. It preferentially binds to CpG island promoters and acts as a transcriptional activator [35]. Meanwhile, Vezf1 is essential for maintaining DNA methylation at various genomic sites. Loss of both copies of *Vezf1* results in widespread demethylation, affecting Line1 elements and minor satellite repeats, some imprinted genes, and CpG islands in mESCs [36]. Notably, *Ssm1b* is expressed in early embryos and promotes CpG methylation in the mouse transgene *HRD* (heavy chain enhancer, rearrangement by deletion) [37]. Finally, DNA hypomethylation at the *ZNF206*-exon 5 CpG island is associated with neuronal differentiation [38]. Collectively, these findings demonstrate that ZFPs construct a sophisticated epigenetic regulatory network through recognizing methylation marks, modulating chromatin accessibility, mediating methyltransferase activity, and preserving genomic stability.

### 3.2. ZFPs and Histone Modifications

**Figure 2:**
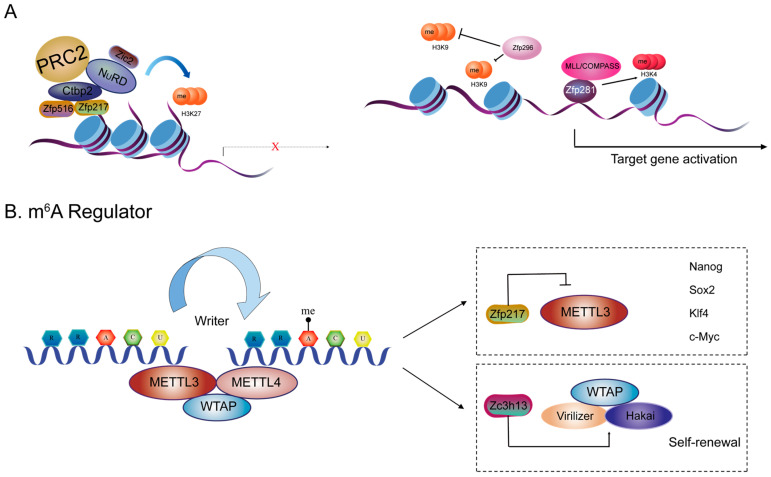
(**A**): ZFPs inhibit or promote gene transcription by recruiting different chromosome modifiers and interacting with different partner proteins. (**B**): ZFPs control the molecular function of m^6^A deposition in ESCs.

ZFPs play a central role in pluripotency and differentiation in ESCs through dynamic regulation of histone modification networks [39,40]. They can either inhibit or promote gene transcription by recruiting various chromatin modifiers and interacting with different partner proteins (Figure 2A). ZFPs exert pivotal regulatory functions during ESC differentiation by mobilizing chromatin-remodeling complexes such as NuRD. For instance, Zfp217/Zfp516 work with Ctbp2 to recruit the NuRD complex, leading to H3K27 deacetylation, thereby establishing a repressive chromatin landscape [39]. Moreover, ZFPs, such as ZBTB2, can interact with GATAD2A/B of the NuRD via their BTB domains during pluripotency withdrawal [41,42]. In addition, Zfp281 can mediate Nanog autorepression by recruiting the NuRD complex [43]. Furthermore, Zic2 colocalizes with the Mbd3-NuRD complex and is essential for the maintenance of H3K27me3 chromatin state and transcriptional repression of the homeotic cluster in mESCs [15].

Many of the promoters of lineage-specific genes in human and mouse ESCs are marked by the active trimethylated histone H3 lysine 4 (H3K4me3) and the repressive trimethylated histone H3 lysine 27 (H3K27me3), known as bivalent domains [44,45]. Durable gene silencing through the formation of compact heterochromatin domains plays a critical role during mammalian development in establishing defined tissues capable of retaining cellular identity. One of the hallmarks of heterochromatin gene repression is trimethylation of lysine 9 on histone H3 (H3K9me3) [46]. Zfp296 negatively regulates H3K9me3 in embryonic development. A knockout of *Zfp296* downregulates early epiblast-marker genes and elevates chromatin accessibility, leading to a unique state of pluripotency in the ESCs [47,48,49]. Most notably when mediating H3K4me3-dependent activation of differentiation genes like *Dnmt3L*, *Lin28a*, and *Foxh1*. Zfp281 achieves chromatin state switching through interaction with the complex of proteins associated with Set1 (COMPASS) without altering overall accessibility, highlighting the functional diversity of ZFPs in gene expression regulation [50]. These interactions illustrate how ZFPs integrate multifaceted chromatin-modifying mechanisms to spatiotemporally orchestrate gene expression networks during ESC fate decisions.

### 3.3. ZFPs and N6-Methyladenosine (m^6^A) Methylation

N6-methyladenosine (m^6^A) methylation is the most common and abundant modification on mammalian messenger RNA (mRNA) and regulates the pluripotency of ESCs [51]. The newly discovered Zfp217 controls the molecular function of m^6^A deposition in ESCs [52]. Supplementation of melatonin during long-term ESC culture enhances the pluripotency of long-term ESCs. It was further found that melatonin promoted the expression of pluripotent factors mainly via Zfp217-dependent m^6^A modification [53]. Scientists employed methylated RNA immunoprecipitation sequencing (MeRIP-Seq) to conduct genome-wide profiling of m^6^A epigenetic modifications in control and *Zfp217* knockdown ESCs. This analysis systematically identified that 3586 m^6^A modification sites increase upon *Zfp217* knockdown. Furthermore, MeRIP revealed a significant elevation in m^6^A modification levels within target RNAs in *Zfp217* knockdown cells, which was associated with a concomitant reduction in the stability of *Nanog*, *Sox2*, *c-Myc*, and *Klf4* mRNAs. Further studies have elucidated the direct involvement of Zfp217 in the transcriptional activation of key pluripotency genes, including Nanog and Sox2. Zfp217 achieves this by interacting with the m^6^A methyltransferase complex component METTL3, sequestering it and thus modulating m^6^A deposition on these crucial transcripts [54]. In addition, LC-MS/MS shows that Zc3h13 plays a critical role in anchoring WTAP, Virilizer, and Hakai in the nucleus to promote m^6^A methylation and regulate mESC self-renewal [55] (Figure 2B).

## 4. ZFPs and ERV Regulatory Network in PSCs

Endogenous retroviruses (ERVs), remnants of ancient retroviral infections integrated into host genomes during evolution, play a crucial role in the silencing machinery of higher vertebrates [56]. KRAB-domain-containing zinc finger proteins (KRAB-ZFPs), the largest subclass of C2H2 zinc finger transcription factors encoded by vertebrate genomes, interact with the corepressor KAP1 (TRIM28/TIF1β) to orchestrate the repression of endogenous retroviral elements. The synergistic action of KRAB-ZFPs, KAP1, and SETDB1 not only directly suppresses ERV activity but also ensures long-term maintenance of genomic integrity [57]. For instance, ZFP932 and Gm15446 target subsets of endogenous retrovirus-K (ERVK) in mESCs [58], while Zfp819 interacts with KAP1 to suppress the activity of ERVs in ESCs [59]. Additionally, KAP1 collaborates with specific KRAB-ZNFs (ZNF114, ZNF483, ZNF589) to silence differentiation-related genes via H3K9me3 and DNA methylation, thereby reshaping the epigenetic landscape [60].

During mESC differentiation, KAP1 is actively recruited to pericentromeric heterochromatin regions [61], and the integrity of its RING/PHD domains is critical for maintaining human iPSC pluripotency [62]. The KZFP/KAP1 complex is pivotal in ESCs, where it anchors to imprinting control regions (ICRs) and transposable elements (TEs) in a sequence-specific manner [63]. For example, ZFP809 binds murine leukemia virus (MLV) DNA elements and recruits KAP1 to repress their activity in mESCs [64,65], whereas KAP1 paradoxically promotes ZFP809 degradation in differentiated cells [66]. ZFP708 [67], Zfp281 [68], and Zfp92 [69] target RMER19B, LINE-1, and B1/Alu SINE transposable elements, respectively, modulating retroelement and gene expression. In hESCs, the KZFP/KAP1 complex controls a broad spectrum of human-specific endogenous retroelements (EREs), with recruitment of KAP1 and DNA methylation exhibiting family-dependent interdependence [70]. YY1 participates in ERV repression by binding to their LTR regions [71], and ZNF91 interaction with the VNTR region of SVA elements restricts their transcriptional activity [72].

## 5. ZFPs in Embryonic Development

Zygotic genome activation (ZGA) marks a crucial stage in embryonic development, characterized by the activation and transcription of paternal and maternal genomes after fertilization, which initiates early embryogenesis [73]. During ZGA, embryos undergo changes in a series of epigenetic events, including whole-gene DNA methylation and histone modifications [74,75]. Interestingly, ZFPs collaboratively construct spatiotemporal-specific gene expression networks necessary for embryonic development by integrating epigenetic signals with chromatin remodeling [48,76,77,78]. Notably, YY1 acts as a critical regulator during ZGA by facilitating nucleosome assembly to modulate chromatin accessibility with its binding sites showing H3K27ac enrichment in 8-cell and morula embryos, indicating active enhancer establishment [79].

ZFPs play pivotal roles in various stages of embryonic development [80,81,82,83]. A recent study showed that *Zfp191*^−/−^ embryos experienced severe developmental delay and died approximately 7.5 days post-fertilization [10]. Additionally, reducing the level of *Zscan4* by siRNAs delayed the progression from the 2-cell stage to the 4-cell stage and prevented blastocysts from implanting or proliferating in blastocyst explant cultures [84]. Specific ZFP members like Zfhx1b [85,86], SALL1 [87], ZNF804A [88], and POGZ [89] are involved in neurodevelopment, while ZFP541 [90] and Rex1 [91] perform essential functions in germ cell meiosis. Furthermore, Zfp800 is predominantly expressed in pancreatic MPCs and endocrine progenitor populations with knockout mice displaying abnormal pancreatic developments [92].

## 6. ZFPs Act as Regulator of PSCs

PSCs are classified as naïve and primed based on their growth characteristics in vitro and their potential to give rise to all somatic lineages and the germ line in chimeras [93]. The transition from a naïve to a formative to a primed state accompanies dynamic changes in self-renewal ability and differentiation potential [94]. In mice, PSCs are thought to exist in a naïve state, which is the cell culture equivalent of the immature pre-implantation embryo, whereas in humans, PSCs are in a primed state, which is a more committed pluripotent state than a naïve state [95]. The acquisition and maintenance of pluripotency are intrinsically related to the core regulatory network of specific transcription factors and are tightly controlled by signaling pathways [96].

### 6.1. ZFPs Act as Regulator of Diverse Pluripotent States

ZFPs play a critical role as bidirectional regulators, orchestrating the delicate balance between the naïve and primed states of PSCs. These proteins determine the cell fate by facilitating transitions and maintaining the integrity of each pluripotent state [97,98]. For instance, Zfp281 functions as a bidirectional regulator of cell state interconversion, promoting exit from naïve pluripotency [99]. It achieves this by inhibiting the expression of genes associated with naïve pluripotency and interacting with Tet1 to drive the transition of mESCs from the naïve-to-primed state [40]. Furthermore, Zfp281 prevents mESCs from transitioning to the 2C-like state [100] and limits the differentiation of ESCs into XENs, thereby maintaining the pluripotency of ESCs [101]. In contrast, depleting Zfp819 in mESCs causes them to transition to a 2C-like state [102]. On the other hand, ZBTB12 acts as a molecular barrier to dedifferentiation in hPSCs; its depletion enhances hPSC self-renewal while promoting dedifferentiation toward a naïve-like state. Mechanistically, ZBTB12 silences long non-coding RNAs (lncRNAs) to drive exit from pluripotency and coordinate orderly three germ layer differentiation [103].

### 6.2. ZFPs Act as Regulators of ES Cell Identity

ZFPs, as key components of the core transcriptional regulatory network, play a crucial role in maintaining stem cell pluripotency and self-renewal. Multiple studies have revealed that various ZFPs are involved in regulating the pluripotent state of stem cells by directly binding to the promoter regions of key pluripotency genes like *Sox2*, *Oct4*, and *Nanog* [7,8,12,13,14,28,29,30,59,104,105,106,107,108,109,110,111,112,113,114,115,116,117,118,119,120,121,122,123,124] (Table 1). Among them, Zscan10 (Zfp206) can activate the promoters of *Oct4* and *Nanog* [104,105]. However, Zscan10 is dispensable for maintaining mESC pluripotency, as *Zscan10* knockout has no significant effect on ESC self-renewal capacity [125]. Similarly, Rex1 (Zfp42) is expressed in various pluripotent cell types [123,126] and has been used as a mark to indicate the undifferentiated state of ESCs [127]. However, conflicting conclusions have emerged regarding the functional roles of Rex-1. When this gene is silenced via RNA interference, ESCs exhibit significantly compromised self-renewal capacity. Paradoxically, overexpression of Rex1 also exerts an inhibitory effect on self-renewal [128,129].

**Figure 3:**
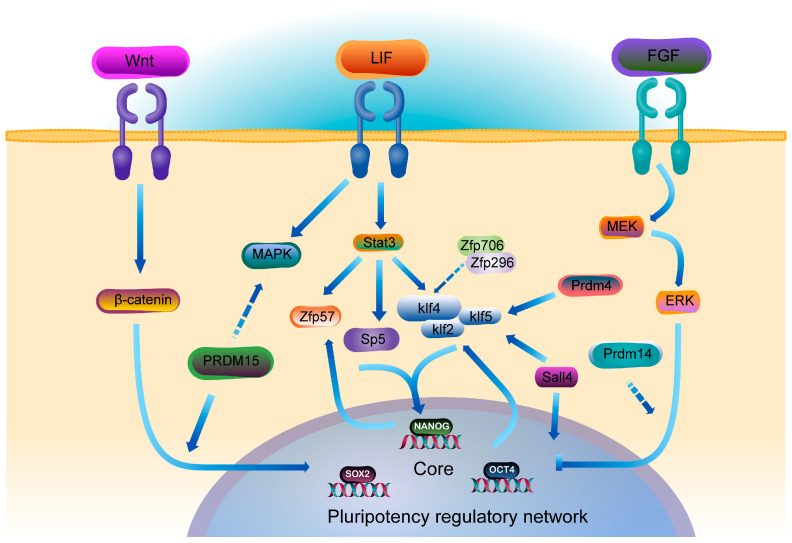
ZFPs are involved in ES cell signaling pathways to affect the pluripotency regulatory network.

ZFPs play a crucial role in the pluripotency regulatory network of ESCs by integrating core signaling pathways such as LIF/Stat3 [111,131]. The Klf transcription factor family (Klf2/4/5) forms a synergistic regulatory network with Nanog, thereby maintaining the self-renewal capacity of mESCs [107,108,109,110]. Among them, the expression of Klf4 is selectively upregulated by the LIF/Stat3 signaling axis [132], while Zfp706/Zfp296 promote the timely exit of ESCs from self-renewal and initiate differentiation by inhibiting Klf4 expression [133,134]. PRDM family members maintain ESC pluripotency through multi-level regulatory mechanisms. Prdm4 acts upstream of Klf5 and is involved in regulating Klf5 function [135]. Recent experiments suggest that Prdm14 plays a pivotal role in maintaining the so-called naïve pluripotency. Specifically, Prdm14 can establish the global DNA hypomethylation signature characteristic of naïve pluripotency by inhibiting DNA methyltransferases (DNMT3A/3B/3L) and the FGF signaling pathway [136,137,138]. PRDM15 further ensures the stability of the naïve pluripotent state by activating the WNT pathway and inhibiting MAPK signaling [139] (Figure 3).

## 7. The Role of ZFPs in Differentiation of PSCs

**Figure 4:**
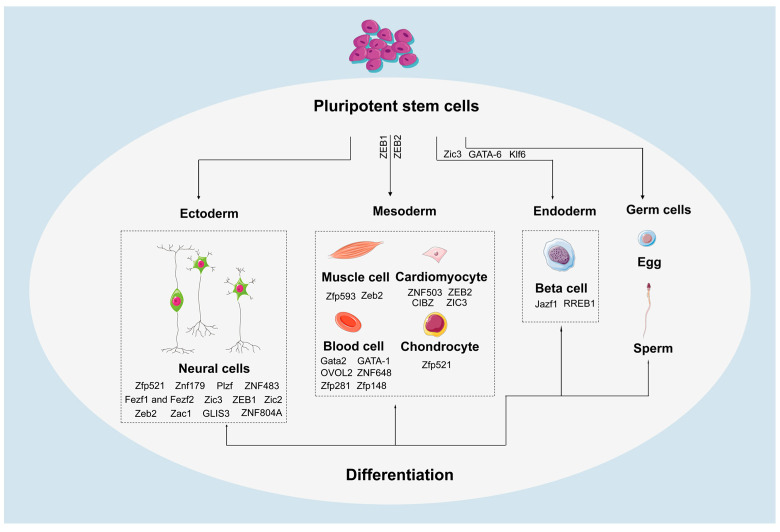
ZFPs play regulatory roles in differentiation of PSCs.

ZFPs play a crucial role in maintaining the balance between self-renewal and differentiation potential of PSCs. They regulate the differentiation trajectories of PSCs towards the three germ layers [140,141,142,143,144,145,146,147] (Figure 4). Firstly, Zfp157 is expressed in the epiblast [143]. Deletion of *Zc3h11a* results in impaired differentiation towards epiblast-like cells [144]. An analysis of the interactions of Zic3 within mESCs has revealed its function in suppressing endodermal differentiation [114]. Additionally, ZFPs also play key roles in specific differentiations, such as promoting cardiac differentiation [148,149,150], regulating skeletal muscle differentiation [151,152], and osteogenic differentiation [153].

ZFPs play a pivotal role in hematopoietic development by precisely orchestrating gene regulatory networks that control the generation of various blood cell lineages. Specifically, these proteins employ multi-layered mechanisms to modulate hematopoietic lineage differentiation [154]. For example, aberrant overexpression of *Gata2* significantly enhances the formation of hemogenic endothelial cells (HECs), highlighting the critical influence of transcription factor levels on cell fate determination [155]. In the process of erythropoiesis, GATA-1 acts as a master regulator [156] since it forms complexes with ZFPs Zfp281 and Zfp148 (ZBP-89) to collectively regulate a set of genes necessary for erythroid cell differentiation [157,158]. Notably, recent studies have revealed that *ZNF648* depletion hinders the differentiation of megakaryocytes and erythroid cells [159], further underscoring the wide-ranging regulatory functions of the ZFPs in hematopoietic development.

ZFPs also play important roles in neural differentiation [15,160,161,162,163,164,165,166,167,168,169], participating in processes such as regulating the cell cycle [162], promoting the expression of neural markers [170,171], and influencing neural progenitor cells (NPCs) in the nervous system [166,172,173,174,175,176,177]. For example, the loss of Zic3 function leads to an increase in neural differentiation markers such as Neurog1 and Her9, indicating its inhibitory role in neural differentiation [170]. Furthermore, Zfp521 is key in maintaining neural differentiation [178], cooperating with p300 to activate early neural marker genes by activating early neural marker genes *Sox1*, *Sox3,* and *Pax6* [171]. Interestingly, the influence of Zfp521 extends to other differentiation pathways, such as chondrocyte proliferation and differentiation [179], as well as bone formation [180].

Consistently, ZFPs exhibit multidimensional regulatory functions in cell fate determination of iPSCs. On one hand, iPSCs derived from schizophrenia patients differentiate into functional glutamatergic neurons expressing ZNF804A. This not only correlates with disease pathogenesis [181] but also regulates neurite outgrowth and dendritic spine morphology through synaptic localization [182]. Knockdown experiments further implicate ZNF804A in modulating neuronal inflammatory responses [183]. On the other hand, metabolic disease studies reveal that iPSCs from *Jazf1* knockout cells showed impaired differentiation into insulin-secreting β-cells, leading to glucose homeostasis dysregulation and type 2 diabetes phenotype [184]. Notably, RREB1 modulates in vitro differentiation of hiPSCs into β-like cells, where its knockout induces NEUROG3 transcriptional activation that significantly accelerates endocrine lineage commitment. This altered expression pattern directly drives an accelerated differentiation trajectory toward the endocrine lineage [185].

## 8. ZFPs in Regulation of Somatic Cell Reprogramming

**Figure 5:**
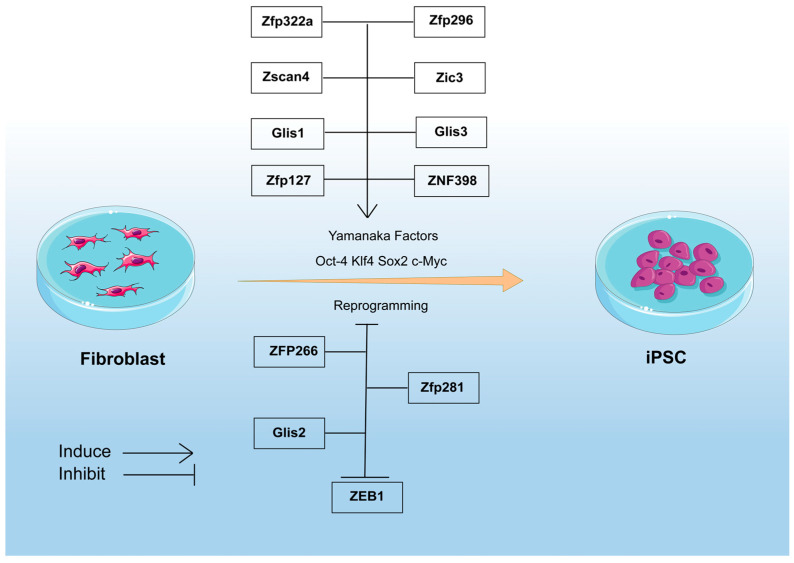
The regulatory role of ZFPs in reprogramming.

Since the groundbreaking discoveries by Yamanaka’s group [186,187] and another independent team [188] that demonstrated the reprogramming of fibroblasts into ESC-like cells using defined transcription factor combinations (OSKM/Yamanaka factors: Oct4, Sox2, Klf4, c-Myc; OSNL/Thomson factors: Oct4, Sox2, Nanog, Lin28), the field of regenerative medicine has undergone revolutionary advancements. Scientists have since experimented with different methods to obtain iPSCs by utilizing different variations of the OSKM cocktail and improve the derivation of iPSCs [189,190,191]. While subsequent studies have made significant progress in optimizing iPSC generation through modified factor combinations, reprogramming efficiency remains constrained by intricate molecular mechanisms. Recent investigations have shed light on the crucial regulatory roles of ZFPs in this process. These proteins can either directly replace traditional transcription factors or establish functional synergistic networks with them, allowing for precise bidirectional modulation of cellular reprogramming efficiency (Figure 5).

### 8.1. ZFPs in Promoting Somatic Cell Reprogramming

ZFPs can act through direct substitution or functional synergy with canonical transcription factors, thereby significantly enhancing both the efficiency and safety of cellular reprogramming processes [192,193,194,195,196,197]. The combinatorial expression of Zscan4 [192], Zfp322a [196], Zfp296 [197], Glis1, and Glis3 [198,199] with the Yamanaka factors synergistically enhances reprogramming efficiency, resulting in a more robust induction of iPSC formation. Knockdown of *Zic3* during OSKM-induced iPSC generation significantly compromises colony formation efficiency [195]. Additionally, Zscan4 not only reactivates early embryonic genes and maintains genomic stability during reprogramming but also enhances iPSC generation efficiency through its family member Zscan4f-mediated metabolic reprogramming and proteasome function enhancement, thereby acting as a critical enhancer in promoting somatic cell reprogramming [193,194]. Our laboratory demonstrates that Zfp322a serves as a novel reprogramming factor capable of substituting Sox2 within the classical Yamanaka cocktail. Remarkably, this factor exhibits synergistic potential when combined with the Yamanaka factors, resulting in a pronounced augmentation of reprogramming efficiency and accelerated onset of the cellular reprogramming process [196]. Meanwhile, Glis1 replaces oncogenic c-Myc while significantly enhancing murine and human fibroblast reprogramming efficiency through early activation of glycolytic metabolic reprogramming. Its reduced oncogenic potential further provides critical safety advantages for clinical-grade iPSC production [200,201,202,203,204].

Furthermore, epigenetic modifications have been recognized as instrumental in enhancing reprogramming efficiency. Zfp127 overexpression in fibroblasts induced demethylation of the *Oct4* promoter, thereby increasing *Oct4* promoter activity, offering higher reprogramming efficiency [205]. Proper reprogramming of epigenetic marks is essential for somatic cells to regain pluripotency. Enhanced in vitro reprogramming into cloned embryos and iPSCs has been observed after the overexpression of Kdm4b in MEFs, which decreases H3K9/36me3 levels and is associated with the upregulation of Zfp37 [206]. Additionally, ZNF398 has been identified as a key player in the reprogramming process. The knockdown of *ZNF398* results in a significant reduction of iPSC colony formation. Mechanically, ZNF398 collaborates with SMAD3 and the histone acetyltransferase EP300 to bind active promoters and enhancers, promoting the transcription of TGF-beta target genes [207].

### 8.2. ZFPs in Blocking Somatic Cell Reprogramming

Recent studies have revealed that not all ZFPs support the generation of iPSCs, with some like Glis2 [198] and Zfp281 [43,99] inhibiting the process. In addition, ZFP266 has been identified as an inhibitor of reprogramming. It binds to short interspersed nuclear elements (SINEs) near the binding sites of the reprogramming precursor factors OCT4 (POU5F1), SOX2, and KLF4, hindering chromatin opening. Remarkably, depletion of Zfp266 significantly boosts iPSC generation in various reprogramming scenarios, highlighting it as a major obstacle. However, ZFP266 was transformed from a suppressor to a potent promoter of iPSC reprogramming by replacing the KRAB co-suppressor with co-activation domains [208].

ZEB1 functions as the E-cadherin repressor and is typically suppressed to aid reprogramming. For instance, the pluripotency regulator NAC1 directly inhibits ZEB1 through transcriptional inhibition, which is essential for iPSC generation [209]. Moreover, reducing ZEB1 also enhances GSC reprogramming [210]. In addition, the miR-200c-141 cluster has been shown to significantly reduce the expression of ZEB1, thereby improving reprogramming efficiency [211].

## 9. The Role of ZFPs in Human Health

In recent years, dysfunction of ZFPs has been demonstrated to participate in the pathogenesis of various diseases by disrupting cellular signal transduction and gene regulatory networks. Representative examples include: Mowat–Wilson syndrome [212], autism spectrum disorder [213], neurodevelopmental disorder [214], autoimmune pathology [215], rheumatoid arthritis [216], osteoarthritis [217], and renal fibrosis [218]. Additionally, ZFPs serve as multifaceted prognostic biomarkers in various cancers linked to DNA damage repair and cell cycle regulation [219,220], immune infiltration [221,222], angiogenesis [223], EMT [224,225], and m^6^A modification [226,227], exhibiting dual oncogenic and tumor-suppressive roles. Clinically, they have been utilized both as therapeutic targets [228] and in combination with chemotherapy to enhance tumor sensitivity. For example, ZNF480 [229] and ZFP64 [230] significantly promote resistance to neoadjuvant chemotherapy and doxorubicin in breast cancer. Respectively, ZNF143 mediates resistance to lenvatinib and sorafenib in hepatocellular carcinoma [231]. ZNF263 enhances the tolerance of colorectal cancer cells to combined chemoradiotherapy (CRT) [232]. Additionally, targeting ZFP64 enhances the therapeutic efficacy of nab-paclitaxel and reverses the immunosuppressive microenvironment in gastric cancer [233]. Furthermore, GLI2 inhibitors demonstrate significant potential in reversing platinum resistance in gastric cancer experimental models, effectively enhancing the chemotherapy sensitivity of tumor cells to cisplatin [234]. These studies suggest that ZFPs play divergent functions which are context dependent. Malfunctions of ZFPs may be associated with different human diseases, highlighting the important role of ZFPs in human health.

## 10. Conclusions and Prospects

PSCs including ESCs and iPSCs have provided unprecedented opportunities for cell-based therapies targeting incurable diseases and injuries [235]. In this article, we reviewed the progress of ZFPs in pluripotent stem cells, including their impact on pluripotency and differentiation through regulating core transcriptional network pathways, as well as their roles in promoting or inhibiting gene expression by regulating epigenetic mechanisms such as DNA methylation and histone modification. ZFPs also play important roles in regulating somatic reprogramming. By understanding the mechanisms through which ZFPs exert their effects, researchers can potentially manipulate pluripotent stem cells for therapeutic purposes, including generating specific cell types for tissue engineering or developing novel strategies for treating degenerative diseases. Furthermore, the emerging work based on CRISPR and zinc finger proteins technology treats diseases by precisely editing disease-causing genes. For instance, scientists have employed the dCas9 protein and ZFPs for targeted localization to Na_v_1.7, thereby inhibiting its expression and alleviating chronic pain in murine models [236]. Despite significant progress, there are still scientific challenges to overcome and opportunities for further research. Firstly, each ZFP is unique and functions in a different way. Hence, further work is required to identify novel ZFPs that are important in pluripotency and reprogramming; secondly, how ZFPs are integrated into the whole ESC transcriptional network and how ZFPs crosstalk with other genetic/epigenetic factors are still unclear; thirdly, biochemical and structural studies might be required to uncover the dynamic interactions between zin finger domains with DNA/RNA/proteins. Nevertheless, with the help of cutting-edge technologies, the comprehensive study of ZFPs will unlock new insights into cellular functions and epigenetic regulation, ultimately advancing biomedical sciences and creating new opportunities for therapeutic applications.

## Figures and Tables

**Table 1:** Summary of pluripotency-associated zinc finger proteins.

Name	Aliases	Role	Species	Target Genes	Mechanism	References
Zfp281	Znf281(mouse) ZBP-99 (human)	mESC	Mouse	Nanog Nanog	Regulate pluripotency by activation and repression of target genes	[7,8]
Patz1	8430401L15Rik, Mazr, Patz, Zfp278 (mouse) MAZR, PATZ, RIAZ, ZBTB19, ZNF278, ZSG, dJ400N23 (human)	mESC	Mouse	Oct4, Nanog	Maintain pluripotency	[12]
Zfp553	2600009K23Rik, C330013F15Rik, Znf48 (mouse)	mESC	Mouse	Oct4, Nanog	Maintain pluripotency	[13]
Zfp143	D7Ertd805e, KRAB14, SBF, Staf, Zfp79, Zfp80-rs1, Znf143, pHZ-1 (mouse)	mESC	Mouse	Oct4, Nanog	Maintain pluripotency	[14]
Sall4	5730441M18Rik, C330011P20Rik, Tex20 (mouse) DRRS, HSAL4, IVIC, ZNF797 (human)	mESC	Mouse	Oct4, Nanog, Sox2	Maintain pluripotency	[28,29,30]
Zfp819	4930427I11Rik, 4933405K07Rik (mouse)	mESC	Mouse	Oct4, Nanog, Sox2	Downregulation of pluripotency marker genes	[59]
Zscan10	Zfp206, Zkscan10, Znf206 (mouse) OFNS, ZFP206, ZNF206 (human)	mESC	Mouse	Oct4, Nanog, Sox2	1, Pluripotency factor 2, No impact on self-renewal	[104,105,106,125]
klf2	Lklf (mouse) LKLF (human)	mESC	Mouse	Nanog	Sustain self-renewal	[107]
klf4	EZF, Gklf, Zie (mouse) EZF, GKLF (human)	mESC hESC	Mouse Human	Nanog	Sustain self-renewal	[107,108]
Klf5	4930520J07Rik, Bteb2, CKLF, IKLF (mouse) BTEB2, CKLF, IKLF (human)	mESC	Mouse	Nanog	Sustain self-renewal	[109,110]
Sp5	-	mESC	Mouse	Nanog	Sustain self-renewal	[111]
Zfp57	G19, Zfp-57 (mouse) C6orf40, TNDM1, ZNF698, bA145L22, bA145L22.2 (human)	mESC	Mouse	Nanog	Downstream target of Nanog	[112]
ZNF207	8430401D15Rik, BuGZ, Zep, Znf207 (mouse) BuGZ, hBuGZ (human)	hESC	Human	OCT4	Required for self-renewal and pluripotency	[113]
Zic3	Bn, Ka (mouse) HTX, HTX1, VACTERLX, ZNF203 (human)	mESC	Mouse	Oct4, Nanog, Sox2	Maintain pluripotency	[114,115]
Sall1	Msal-3 (mouse) HEL-S-89, HSAL1, Sal-1, TBS, ZNF794 (human)	mESC	Mouse	Nanog, Sox2	Regulate pluripotency	[116]
Zfp462	6030417H05, 9430078C22Rik, Gt4-2, Zfpip, Znf462 (mouse) WSKA, ZFPIP, Zfp462 (human)	P19	Mouse	Oct4, Nanog, Sox2	Maintain pluripotency	[117,118]
Zscan4c	Gm397, XM_142517, Zscan4d (mouse)	mESC	Mouse	-	Regulator of pluripotency	[119]
YY2	ZNF631 (human)	mESC	Mouse	Zscan4 Oct4	Required for self-renewal	[120,130]
ZFX	Zfx55,6, Zfx6, Zfx (mouse) MRXS37, ZNF926 (human)	mESC hESC	Mouse Human	Tbx3, Tcl1	Promote self-renewal	[121,122]
Zfp42	Rex-1, Rex1, Zfp-42 (mouse) REX-1, REX1, ZNF754, zfp-42 (human)	mESC hESCF9	Mouse Human	-	Pluripotency marker	[123,124,127,128,129]
